# Improving the ability to discriminate medical multiple-choice questions through the analysis of the competitive examination to assign residency positions in Spain

**DOI:** 10.1186/s12909-024-05324-2

**Published:** 2024-04-03

**Authors:** Eduardo Murias Quintana, José Rodríguez Castro, Fernando Sánchez Lasheras, Juan Vega Villar, Jose Juan Curbelo García, María Cadenas Rodríguez, Jaime Baladrón Romero

**Affiliations:** 1 Department of Radiology, Hospital Universitario Central de Asturias, Avenida de Roma S/N, Oviedo, Asturias 33011 Spain; 2 Department of Mathematics, University of Oviedo, Oviedo, Spain; 3 ”Curso Intensivo MIR Asturias” Academy, Avenida de Roma S/N, Oviedo, 33011 Spain

## Abstract

**Introduction:**

Psychometrics plays a vital role in evaluating educational research, including the analysis of multiple-choice exams. This study aims to improve the discriminatory ability of the “Médico Interno Residente” (MIR) medical exam in Spain, used to rank candidates for specialized healthcare training, through psychometric analysis.

**Methods:**

We analyzed 2,890 MIR exam questions from 2009 to 2021 (totaling 147,214 exams), categorizing them based on methodology and response type. Evaluation employed classical test theory and item response theory (IRT). Classical test theory determined difficulty and discrimination indices, while IRT assessed the relationship between knowledge levels and question performance.

**Results:**

Question distribution varied across categories and years. Frequently addressed knowledge areas included various medical specialties. Non-image-associated clinical cases were the easiest, while case-based clinical questions exhibited the highest discriminatory capacity, differing significantly from image-based case or negative questions. High-quality questions without images had longer stems but shorter answer choices. Adding images reduced discriminatory power and question difficulty, with image-based questions being easier. Clinical cases with images had shorter stems and longer answer choices.

**Conclusions:**

For improved exam performance, we recommend using a clinical case format followed by direct short-answer questions. Questions should be of low difficulty, providing clear and specific answers based on scientific evidence and avoiding ambiguity. Typical clinical cases with key characteristic features should be presented, excluding uncertain boundaries of medical knowledge. Questions should have lengthy stems and concise answer choices, minimizing speculation. If images are used, they should be typical, clear, consistent with the exam, and presented within clinical cases using clinical semiotics and propaedeutics.

## Introduction

Psychometrics is a powerful and intuitive tool that finds extensive applications in the field of education [1]. Its usage spans across various domains, including educational research, where it is closely linked to evaluating teaching competencies and learning processes facilitated by electronic media [2]. Additionally, psychometrics has been an integral part of teacher training programs, incorporating new technologies since the 1990s [3]. Moreover, it plays a significant role in ongoing studies exploring the utilization of digital technologies as educational resources within a university setting [4, 5].

Psychometric techniques have proven invaluable in the evaluation of multiple-choice exams, allowing for the assessment of question quality, internal consistency, discriminatory capacity, and difficulty levels. For many years, we have relied on these psychometric techniques and adapted them through electronic response analysis to enhance student training for the medical residency exam in Spain. Our primary objective is to ensure the utmost quality in exam preparation tests, identify areas where additional training is required, and highlight key concepts that warrant emphasis during post-university training.

The “Médico Interno Residente” medical exam (MIR) aims to rank candidates based on their exam scores and grade point averages, enabling a systematic selection process for specialized healthcare training positions in Spain. This ranking system ensures that placements are offered in an organized manner each year [6, 7]. We designate the exams by the date of the call, which is usually the year prior to their administration. Therefore, the MIR exam for 2022 was conducted in January 2023.

The MIR exam is held annually since 1978 by the Ministry of Health and the Ministry of Education in Spain. It takes place on the same day and at the same time across the country. In the period from 2009 to 2018, the exam consisted of 225 multiple-choice - single select questions, with an additional 10 reserve questions covering any field of medicine. Candidates were given a maximum of 5 h to complete the exam. Each correct answer earned three points, while each incorrect answer resulted in a deduction of one point. In the 2019 and 2020 exams, the number of questions was reduced to 175, plus 10 reserve questions, and the duration of the exam was shortened to 4 h. These changes were not due to the COVID-19 pandemic but were intentional changes in the exam structure made by the Ministry of Health. In the two most recent exams (2021 and 2022), there were 200 questions with 10 reserve questions, and the allotted time was 4 and a half hours [8–13].

Since the 2009 MIR exam, the inclusion of questions associated with one or more images has become a regular practice. These images can be either radiological, referring to diagnostic imaging tests, or non-radiological, encompassing medical records, histology imaging, diagrams, spirometry imaging, electrocardiograms, and so on. The purpose of this study is to analyze the main parameters that influence the discriminatory ability of multiple-choice - single select questions [13]. To achieve this, a set of questions gathered from MIR exams spanning a thirteen-year period will be examined.

## Materials and methods

For our analysis, we compiled questions from the 2009 to 2021 MIR exam sessions, totaling 2,890 questions. In the years analyzed, from the 2009 call to the 2021 call, a total of 147,214 candidates have been analyzed with 147,214 exams, averaging 11,324.2 exams per year. To facilitate our analysis, we classified the multiple-choice - single select questions into different sub-categories [13].

### Regarding the question methodology

1. Case reports without an image: These multiple-choice - single select questions present detailed descriptions instead of images. They require differential diagnoses, treatment decisions, or the diagnostic and therapeutic management of patients based on their medical records, clinical examinations, and laboratory or complementary test results. If imaging tests are mentioned, they are described within the question text.
2. Case reports with an image: These case report-type questions include imaging tests as part of the question.
3. Negative questions: These questions ask the exam taker to identify the incorrect response among the provided options.
4. Multiple-choice - single select questions: This category includes all other questions that are concise and not case reports or negative questions. Typically, these questions are straightforward and require the exam taker to choose the correct response among the options.

### Regarding the response methodology

1. Clinical questions: These questions assess knowledge of medical propaedeutics or clinical symptoms.
2. Etiology questions: These questions pertain to the etiology of specific diseases.
3. Pathophysiology questions: These questions focus on understanding the mechanisms and processes underlying diseases.
4. Diagnostic methods questions: These questions relate to specific diagnostic tests or methods.
5. Treatment questions: These questions involve different modalities of treatment.
6. Others: This category includes questions that don’t fit into the aforementioned response methodologies.

### Additional variables collected for question classification were

1. Type of clinical case with images: Clinical cases with images were categorized based on the specific area of knowledge associated with the image.
2. Imaging technique: Clinical cases with images were further classified based on the specific imaging technique used.
3. Semiology or direct diagnosis: Questions related to radiological and nuclear medicine images were divided into two main groups based on whether they required a direct diagnosis from the image or focused on interpreting clinical signs.
4. Number of characters: The character count of each question was recorded.
5. Number of images in each question.

Psychometrics is the scientific field encompassing various methodologies, techniques, and theories aimed at quantifying and measuring psychological variables within the human psyche. It entails test theory, construction, and the application of reliable and valid measurement procedures. Statistical analysis plays a crucial role in assessing the validity of tests for measuring predefined psychological variables.

When evaluating responses to multiple-choice - single select questions, several psychometric models have been adapted to establish accurate models of each subject’s knowledge level based on the characteristics of the test questions. Two mathematical models, namely classical test theory and item response theory (IRT), were utilized in evaluating the MIR exam. These models have been previously employed and validated in studies focusing on the MIR exam.

Classical test theory enables the measurement of question difficulty, discriminatory capacity, and overall quality based on the number of individuals answering the question and their level of knowledge, as indicated by their final exam score. The following tools are utilized within this framework:

1. Difficulty index (DI) calculation: This index represents the percentage of exam takers who answered the question correctly. Questions can be classified as easy, moderate, or difficult based on the percentage of correct responses.
2. Corrected difficulty index (cDI): This index considers the likelihood of guessing the correct answer and penalizes incorrect responses. The difficulty levels are classified as follows for values ranging from − 0.33 to 0: very difficult, between 0 and 0.33: difficult, between 0.33 and 0.66: optimal, between 0.66 and 0.80: easy, and above 0.80 up to 1: very easy.
3. Discrimination index calculation: This index measures the correlation between exam takers’ overall scores and their scores on specific questions. The point biserial correlation coefficient (rpbis) was used in this study to evaluate the discriminatory quality of questions. This index allows classifying question discrimination as follows: excellent (greater than or equal to 0.40), good (greater than or equal to 0.30 and less than 0.40), fair (greater than or equal to 0.20 and less than 0.30), poor (greater than or equal to 0 and less than 0.20), and very poor (negative).

Item response theory (IRT) is a psychometric theory used to predict how exam takers would respond to questions based on their knowledge levels. Probability models estimate the likelihood of an individual answering a question correctly. In this study, the two-parameter logistic (2-PL) model was employed to assess the relationship between exam takers’ knowledge levels and their likelihood of answering questions correctly. The model considers the difficulty and discriminatory capacity parameters of each question and the subject’s knowledge level. The IRT model includes two values:

1. IRT difficulty: This score represents the question’s difficulty, adjusted for the exam taker’s knowledge level.
2. IRT discrimination (DC-R): This score represents the question’s discriminatory capacity, adjusted for the exam takers’ knowledge levels. Questions are classified as excellent, good, fair, poor, or terrible based on the discrimination coefficient.

The IRT variables allow for the generation of a probability curve illustrating the likelihood of answering a specific test question correctly based on the exam taker’s knowledge level. This curve demonstrates not only the question’s discriminatory ability but also the knowledge level at which maximum discrimination occurs.

Continuous variables are summarized using means, standard deviations, range, and medians. Due to the lack of normality, the comparisons among continuous variables are conducted through the non-parametric Kruskal-Wallis test. Categorical variables are described using absolute and relative frequencies. The interrelationship between categorical variables is assessed using the chi-squared test. P-values below 0.05 are deemed statistically significant and a power value of 0.8 is considered adequate for this research.

When questions are classified by images, 4 different groups are considered (clinical imaging, graphics, pathology and radiology and nuclear medicine) the smallest one (pathology) has 41 questions, which means that for a significance level of 0.05 and considering a power of 0.8 the effect size value is 0.2601. According to *Cohen* [14] with such value it is possible to detect medium effect size differences. In the case of the analysis of questions divided by technique (scintigraphy, no radiological, PET, RM, simple radiology, TC and ultrasound), the smallest groups (scintigraphy and PET) had 5 questions; taking into account a significance level of 0.05 and a power of 0.8, the effect size value is 0.6355 wich means that it is possible to detect large effect size differences. Finally in the case when images are divided in three categories (no radiological, simple radiology and TC) taking into account image technique group, the smallest of these three groups is formed by 63 questions which give us an effect size value of 0.2276, able to detect medium effect sizes also for a significance level of 0.05 and a power of 0.8.

All methods were carried out in accordance with relevant guidelines and regulations. The data obtained belong to a set of public exam results templates made available by the Ministry of Health. These templates are accessible through a free access platform. All participants willingly signed informed consent when registering for the exams, acknowledging that their data will be published anonymously on this platform. The ethics committee of University of Oviedo approved the study since it does not have any ethical conflicts.

## Results

The results of the database analysis are presented, comprising a total of 2,890 questions. These questions correspond to the MIR exams conducted between 2009 and 2021. Regarding the knowledge areas covered in the MIR exam, the most frequently asked ones are Biostatistics, Preventive Medicine, and Public Health (8.2%), followed by Digestive System Diseases (8.1%), Pneumology (6.3%), Cardiology, Infectious Diseases, Nephrology, Gynecology and Obstetrics, and Neurology (5% each), and Pediatrics and Endocrinology with 4.5% of the questions each.

In the Table 1 presents the distribution of the questions subject to analysis by category and year. In other words, the figure allows us to observe the proportion of questions in each exam by category. Table 2*captures the distribution of analyzed questions by question type and year. The weight of clinical cases varies between 10.92% of the total questions in 2009 and 15.28% in 2017 across different calls.*

**Table 1:** Distribution of analyzed questions by category and year

Year	Clinical	Etiology	Pathophysiology	Diagnostic Method	Others	Treatment	NA	Total
*2009*	*46*	*15*	*9*	*54*	*53*	*51*	*1*	*229*
*2010*	*31*	*16*	*6*	*65*	*50*	*65*	*1*	*234*
*2011*	*38*	*17*	*7*	*58*	*43*	*69*	*2*	*234*
*2012*	*38*	*13*	*8*	*65*	*44*	*67*	*0*	*235*
*2013*	*33*	*17*	*11*	*71*	*41*	*60*	*0*	*233*
*2014*	*36*	*19*	*12*	*59*	*41*	*63*	*3*	*233*
*2015*	*47*	*23*	*11*	*53*	*43*	*53*	*0*	*230*
*2016*	*43*	*11*	*9*	*65*	*45*	*59*	*0*	*232*
*2017*	*37*	*8*	*7*	*66*	*50*	*60*	*1*	*229*
*2018*	*34*	*27*	*10*	*48*	*53*	*58*	*0*	*230*
*2019*	*25*	*18*	*13*	*44*	*28*	*53*	*0*	*181*
*2020*	*46*	*16*	*11*	*33*	*24*	*53*	*0*	*183*
*2021*	*48*	*8*	*17*	*58*	*19*	*57*	*0*	*207*
*Total*	*502*	*208*	*131*	*739*	*534*	*768*	*8*	*2890*
*%*	*17.4%*	*7.2%*	*4.5%*	*25.6%*	*18.5%*	*26.6%*	*0.3%*	*100%*

**Table 2:** Distribution of analyzed questions by question type and year, expressed as percentages

Question Type	2009	2010	2011	2012	2013	2014	2015	2016	2017	2018	2019	2020	2021	Total	%
Clinical Case	92	115	107	112	113	108	119	108	95	112	77	68	100	1236	42.8%
CC with Image	25	30	30	34	34	34	30	32	35	33	26	25	25	393	13.6%
Negative	36	24	27	37	32	32	27	37	42	50	33	33	35	445	15.4%
Test	76	65	70	52	54	59	54	55	57	35	45	57	47	726	25.1%
Total	229	234	234	235	233	233	230	232	229	230	181	183	207	2890	100%

In the Table 3 presents the mean values, standard deviations, and medians of the variables iDifCorr, rbpis, and discrimination according to the IRT for different question types. Due to the non-normality of the three variables (iDifCorr, rpbis, and Discrimination R), non-parametric comparison tests, specifically the Kruskal-Wallis test, were conducted. The results obtained indicate statistically significant differences among variables across question types for both iDifCorr, rpbis, and Discrimination measured by the two-parameter item response theory model.

**Table 3:** Mean values, standard deviations, and medians of the variables iDifCorr, rpbis, and Discrimination R for different question types

Question Type	N	Mean iDifCorr	Std. Dev iDifCorr	Median iDifCorr	Mean rpbis	Std. Dev rpbis	Median rpbis	Mean Discrimination R	Std. Dev Discrimination R	Median Discrimination R
Clinical Case	1326	0.5758	0.2733	0.6377	0.3074	0.1279	0.3225	0.8028	0.469	0.752
CC with Image	393	0.5281	0.2937	0.5754	0.2613	0.1103	0.2689	0.6355	0.3542	0.6033
Negative	445	0.5102	0.2973	0.5465	0.2891	0.1319	0.2951	0.7341	0.4474	0.6712
Test	726	0.5255	0.3009	0.580	0.2963	0.1274	0.3127	0.7752	0.442	0.7422
Total	2890	0.5466	0.2881	0.6083	0.2955	0.127	0.3075	0.7625	0.4481	0.7115

The Table 4 shows results of the non-parametric Kruskal-Wallis test for iDifCorr, for rpbis and for discrimination measured by the two-parameter item response theory model. Continuing with the analysis, the number of characters in both the question stem and the distractors, as well as the total number of characters, are examined in relation to the question type. Due to the lack of normality in these variables, the non-parametric Kruskal-Wallis test is utilized (Table 4).

**Table 4:** The statistical analysis is shown based on the question type of the variables of corrected difficulty by chance (IDiffCor), question discrimination according to the biserial correlation coefficient (rpbis), item response theory, and finally the number of characters in the statement, responses, and overall question characters. It can be observed that clinical cases associated with images and negative questions exhibit high difficulty and poorer discrimination compared to other questions. Regarding characters, questions with longer statements and shorter answers demonstrate better discrimination. Typical clinical cases and questions that exhibit better discrimination fulfill these criteria for character distribution

Corrected difficulty index: Kruskal-Wallis Test H = 22.69 DF = 3 *P* < 0.001.				
**Question Type**	**N**	**Median**	**Ave Rank**	**Z**
Clinical Case	1326	0.6377	1523.8	4.65
CC with Image	393	0.5754	1392.3	-1.36
Negative	445	0.5465	1343.9	-2.79
Test	726	0.5800	1393.5	-1.94
Overall	2890		1445.5	
The point biserial correlation coefficient: Kruskal-Wallis Test on rpbis H = 52.22 DF = 3 *P* < 0.001.				
**Question Type**	**N**	**Median**	**Ave Rank**	**Z**
Clinical Case	1326	0.3225	1529.9	5.01
CC with Image	393	0.2689	1188.3	-6.57
Negative	445	0.2951	1402.8	-1.17
Test	726	0.3127	1456.7	0.42
Overall	2890		1445.5	
Discrimination measured by the two-parameter item response theory model: H = 44.13 DF = 3 *P* < 0.001				
**Question Type**	**N**	**Median**	**Ave Rank**	**Z**
Clinical Case	1326	0.7520	1517.1	4.25
CC with Image	393	0.6033	1210.5	-6.01
Negative	445	0.6712	1387.9	-1.58
Test	726	0.7422	1477.2	1.18
Overall	2890		1445.5	
Variable characters stem: Kruskal-Wallis Test H = 1634.79 DF = 3 *P* < 0.001				
**Question Type**	**N**	**Median**	**Ave Rank**	**Z**
Clinical Case	1326	390.0	2044.2	35.52
CC with Image	393	309.0	1641.4	5.01
Negative	445	104.0	715.2	-20.07
Test	726	106.0	693.6	−28.06
Overall	2890		1445.5	
Variable characters answers: H = 20.24 DF = 3 *P* < 0.001.				
**Question Type**	**N**	**Median**	**Ave Rank**	**Z**
Clinical Case	1326	171.0	1419.1	-1.57
CC with Image	393	156.0	1315.0	-3.34
Negative	445	216.0	1531.9	2.37
Test	726	208.0	1511.4	2.46
Overall	2890		1445.5	
Variable total characters: H = 742.74 DF = 3 *P* < 0.001				
**Question Type**	**N**	**Median**	**Ave Rank**	**Z**
Clinical Case	1326	613.5	1858.4	24.49
CC with Image	393	510.0	1529.4	2.15
Negative	445	347.0	979.6	-12.81
Test	726	341.0	931.6	-19.18
Overall	2890		1445.5	

Regarding the clinical cases associated with medical images, their presence has varied between 25 and 34 questions since the 2009 exam (Table 2). Among the medical specialties most associated with an image, pneumology (14.5%), cardiology (11%), and digestive system pathology (10.4%) are the most frequently asked. In terms of imaging diagnosis, 57.9% of the exam’s images correspond to the specialty of radiology and nuclear medicine, with 22.6% being clinical images and a total of 5.4% being pathological anatomy images. Among the types of tests asked within the field of radiology and nuclear medicine, 47.5% are X-ray images, 23.5% are CT scans, 11.5% are ultrasound images, and 2.5% are PET-CT images. As for the type of radiological concept being asked, 80.2% of the questions involve images within a clinical case, while the remaining 19.8% are direct questions about semiology.

## Discussion

The entrance examination for medical residency positions in Spain provides a perfect setting for analyzing multiple-choice - single select medical questions with multiple responses. This is due to its intrinsic characteristics, including the stability of the exam format, the consistency of question types, and the structure of the response options and distractors. Additionally, the sample population is highly homogeneous, consisting mainly of medical graduates from Spanish medical schools. From the exam conducted in 2009 to the one held in 2021, the key characteristics of these questions and their psychometric performance in terms of discrimination and difficulty have been thoroughly analyzed. The objective is to identify the distinctive features of the questions and utilize this analysis to enhance the quality of test design in the field of medicine, aiming for greater effectiveness and precision.

Except for a small variation in the 2019 and 2020 exams, the number of questions remains around 220, resulting in a total of 2890 analyzed questions. When examining the areas of knowledge, abdominal pathology, medical specialties, and biostatistics and preventive medicine are the most asked topics.

From a perspective of difficulty, as measured by the index of difficulty corrected by chance, the easiest questions are non-image-associated clinical cases. These questions involve inquiring about a specific disease, its management, or its diagnosis, providing the examinee with patient anamnesis and diagnostic data. Questions that introduce an image have a higher level of difficulty, and the most challenging questions are negative questions where one must identify the incorrect option among the possible answers. If we analyze the discriminatory power of the questions, as measured by the rpbis analysis and Discrimination R, it is the case-based clinical questions that show the highest discriminatory capacity, with significant differences compared to image-based case questions or negative questions. From this, we can infer that asking highly difficult questions can decrease the discriminatory power of the exam, making it more unfair in psychometric terms by introducing noise in the distribution of the candidates’ scores.

**Fig. 1:**
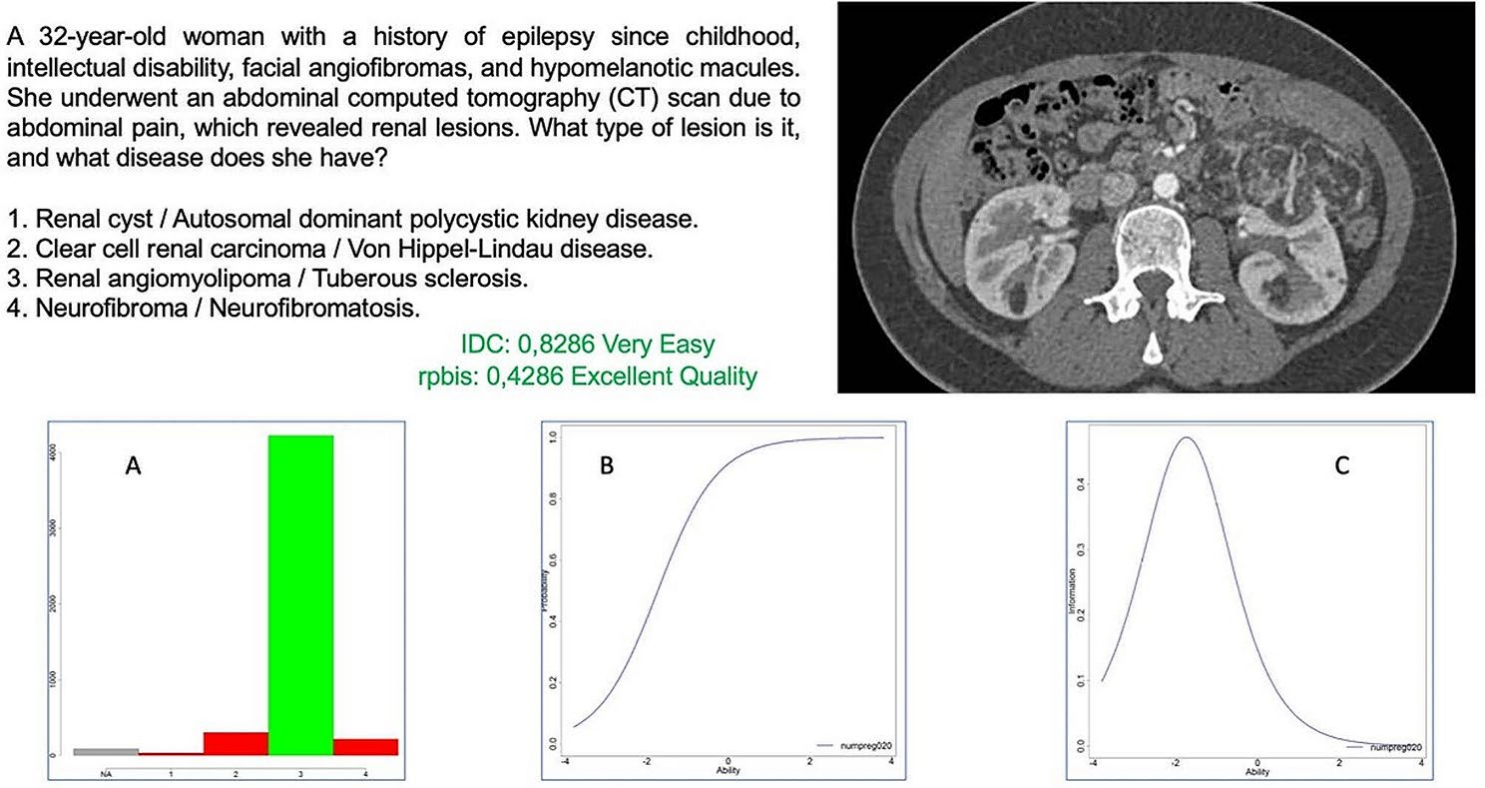
Example of a clinical case question associated with a radiological image. Graph A shows the students’ responses, while graph B represents the distribution of students according to their probability of answering the question correctly (y-axis) based on their ability level in the exam (x-axis). “Ability” refers to the theoretical estimation of the student’s knowledge in the exam. Graph C represents the point at which this question best discriminates among the knowledge levels of the entire sample (x-axis). Both graphs belong to the Item Response Theory (IRT) using the Two-Parameter Logistic (2-PL) probability model. In this case, the question demonstrates excellent quality (rpbis 0.4286) due to a well-crafted and comprehensive statement, concise and precise answers, a typical image relevant to the clinical scenario being queried, and clear instructions in the statement indicating where the student should focus to avoid vague interpretations of other findings. Furthermore, since the concept being assessed is specific, with an adequate scope in the field of medicine and sufficient scientific evidence beyond any subjective interpretation, an excellent quality is achieved. The 2PL probability model demonstrate how students in the strong group perform better than those in the weak group (B), saturating the curve at a knowledge level close to 30% of the overall distribution of knowledge in the exam (C)

Regarding the number of characters in high-quality questions within the exam, clinical cases without images have a higher median number of characters in the stem compared to the rest, and a statistically significant lower median number of characters in the answer choices. These characteristics are crucial for achieving good discriminatory power (Fig. 1). On one hand, providing all the necessary clinical data to the candidates ensures that they have complete information to answer the question, avoiding working with partial or biased data. On the other hand, having concise and specific answer choices with fewer characters reduces ambiguity, subjective interpretation, and subjective data. As a result, the discriminatory power increases, making these questions of higher quality.

**Fig. 2:**
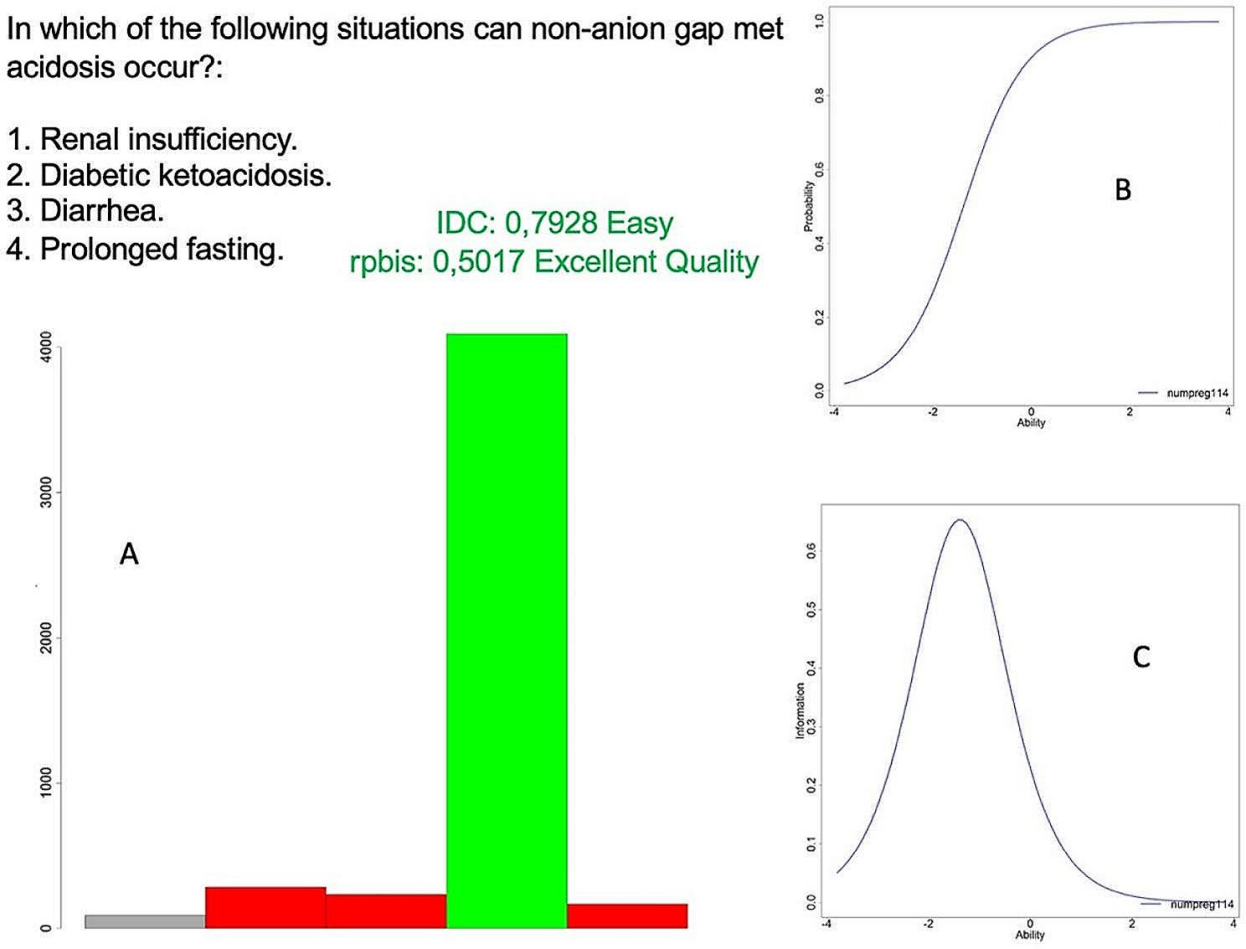
Example of a clinical case question associated with a radiological image. Graph A displays the students’ responses, while graph B represents the distribution of students according to the probability of answering the question correctly (y-axis) based on their ability level in the exam (x-axis). “Ability” refers to the theoretical estimation of the student’s knowledge in the exam. Graph C represents the point at which this question best discriminates among the knowledge levels of the entire sample (x-axis). Both graphs belong to the Item Response Theory (IRT) using the Two-Parameter Logistic (2-PL) probability model. As a typical example of a well-discriminating multiple-choice - single select question, we can observe how it presents a short and direct statement. The question addresses a clear and precise medical concept. The answer choices consist of few characters and are devoid of subjectivity. In this way, these questions follow a “know or don’t know” format, which makes them highly effective in achieving proper discrimination

Direct multiple-choice - single select questions (Fig. 2) exhibit high quality and rank second highest in the exam, following non-image clinical cases. This is because these questions typically involve a direct and specific concept with a clear statement and concise answers of few characters, which are less prone to subjectivity. In this way, these questions follow a “know or don’t know” format, which makes them highly effective in achieving proper discrimination.

**Fig. 3:**
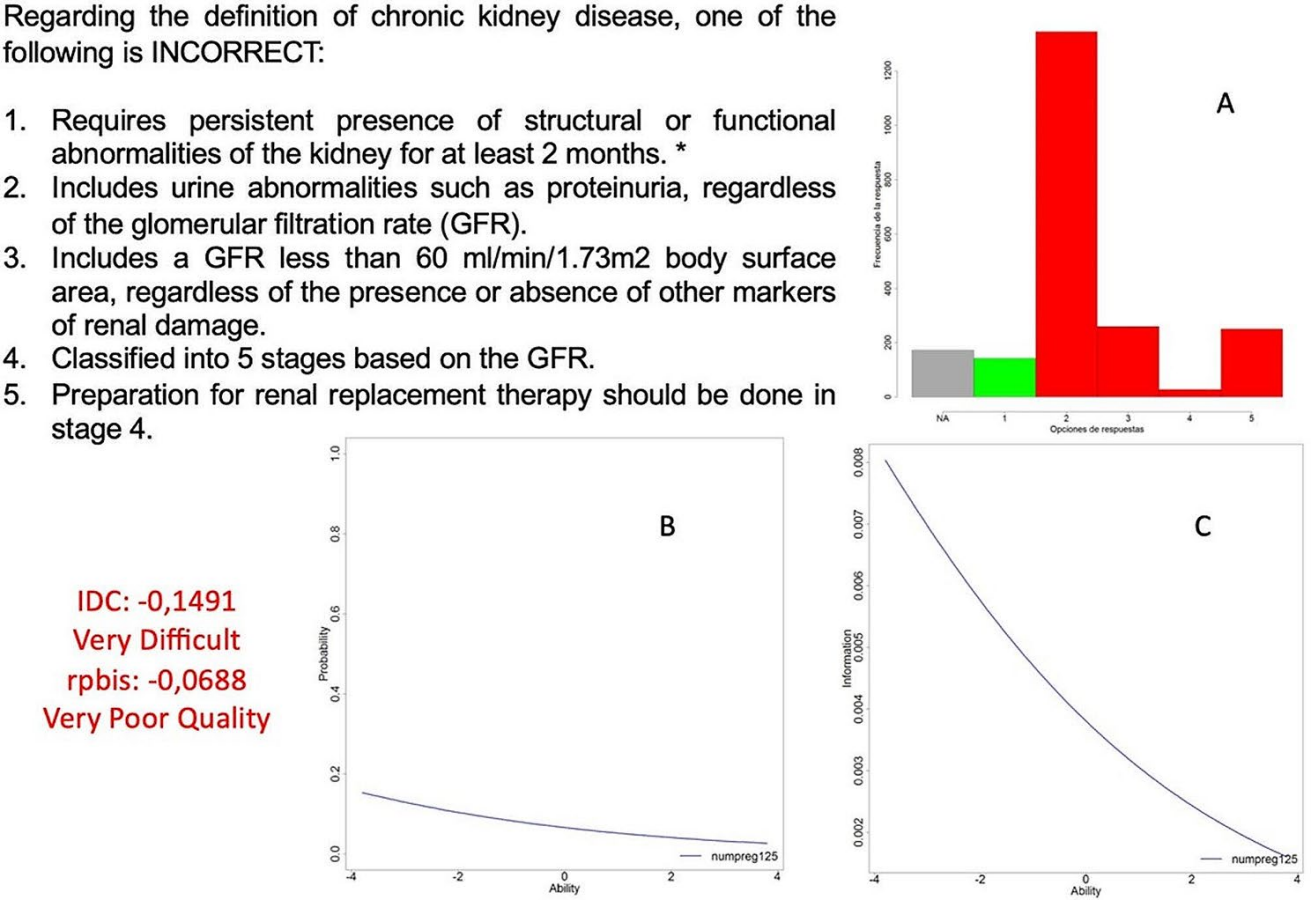
Example of a clinical case question associated with a radiological image. Graph A displays the students’ responses, while graph B represents the distribution of students according to the probability of answering the question correctly (y-axis) based on their ability level in the exam (x-axis). “Ability” refers to the theoretical estimation of the student’s knowledge in the exam. Graph C represents the point at which this question best discriminates among the knowledge levels of the entire sample (x-axis). Both graphs belong to the Item Response Theory (IRT) using the Two-Parameter Logistic (2-PL) probability model. Incorrect questions are technically challenging to construct. In the displayed question, to obtain an incorrect response as the first option, one must rely on a subtle and minor nuance related to a short time frame (not two months, but three months). The remaining correct answers with subjective nuances not only hinder question discrimination but also, as observed in the 2PL model, lead to a situation where students who know more perform worse than those who know less (graph B). This not only introduces noise in the sample but also significantly decreases the overall discrimination of the entire exam

The questions with lower discriminatory power are the negative (Fig. 3), which have shorter stems and longer answer choices. As mentioned before, this leads to greater subjective interpretation of the correct option and less information provided to answer the question, resulting in increased difficulty and lower discriminatory power. Out of the 445 negative questions evaluated, only half of them achieve adequate discrimination. This is related to the described characteristics of both the question text and the answer choices, as well as the technical difficulty involved in developing this type of question. This is because, due to the intrinsic nature of medicine, it is much more challenging to provide a response that is entirely correct than one that is incorrect. In a negative question, one must develop multiple entirely correct answer choices, while the remaining questions have only one correct answer. This technical difficulty makes it very challenging to obtain negative questions that effectively discriminate, and they should be avoided in tests.

It is very challenging to assess medical knowledge and skills related to communication, common sense, or synthesis through multiple-choice - single select questions, as these abilities are encountered daily in routine clinical practice. This does not imply that assessing students’ knowledge in these areas should be neglected, but rather alternative tools should be considered. Multiple-choice - single select questions are suitable for evaluating scientific-technical and theoretical knowledge skills in ideal cases of low difficulty. However, creating multiple-choice - single select questions to assess other aspects of clinical practice is too complex to be cost-effective from a psychometric discrimination standpoint.

**Fig. 4:**
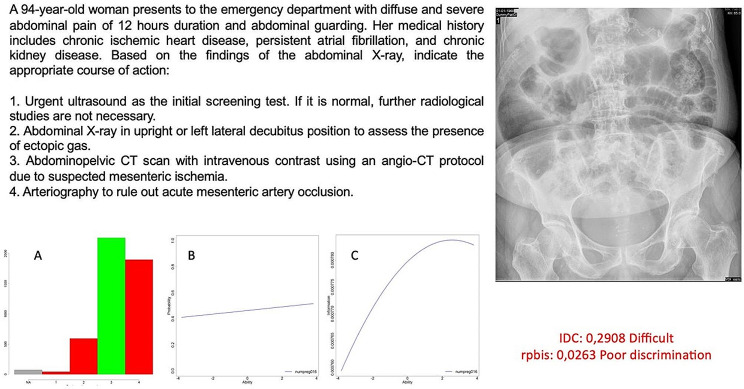
Example of a clinical case question associated with a radiological image. Graph A displays the students’ responses, while graph B represents the distribution of students according to the probability of answering the question correctly (y-axis) based on their ability level in the exam (x-axis). “Ability” refers to the theoretical estimation of the student’s knowledge in the exam. Graph C represents the point at which this question best discriminates among the knowledge levels of the entire sample (x-axis). Both graphs belong to the Item Response Theory (IRT) using the Two-Parameter Logistic (2-PL) probability model. This question exemplifies how errors in its design result in poor discrimination, rendering it inadequate for assessing students’ knowledge. The clinical description is insufficient and too nonspecific to effectively evaluate students’ knowledge. In this case, although it may represent a real-world scenario, better discrimination results are obtained in exams with “ideal” or “typical” cases. Attempting to assess scenarios that are not theoretical, scientific, or supported by clear scientific evidence is not cost-effective with multiple-choice - single select questions. Furthermore, the answers are lengthy, imprecise, subjective in nature, and lacking clear scientific evidence, as they may vary among different hospital protocols. Additionally, the image, while once again typical of routine medical practice in the setting of acute abdomen, does not depict a specific radiological finding but rather a common image associated with numerous pathologies that do not contribute to the diagnosis of a specific condition. With these methodological errors according to the 2PL model, graph B demonstrates how there are no differences between students with varying levels of knowledge when faced with the question

If we analyze the clinical cases associated with images to try to understand why associating an image with a clinical case significantly worsens the discriminatory power, we find the following. For a sample of 393 questions associated with clinical cases with images, the difficulty of these questions is significantly higher compared to clinical cases without images or test questions (Fig. 4). Additionally, clinical cases with images have fewer characters in the stem and more characters in the answer choices.

If we examine the type of concept being asked, despite all the images being diagnostic tests, 80% of them did not ask about the semiotics of what was seen in the image, meaning they did not inquire about the technique’s semiotics. Instead, they asked about clinical elements or treatment of the disease. This requires the candidate not only to diagnose the disease but also to infer its management based on an imaging technique. This directly leads to a significant increase in difficulty, an increase in subjectivity in management, and a decrease in the discriminatory power of the questions. Furthermore, probably due to the type of image being asked, as multiple images of advanced diagnostic techniques have been included, there is a bias towards increased difficulty, resulting in poor discriminatory power, bordering on being considered as having terrible discriminatory power.

The standard practice is to have one image per question, but it is not the case in all instances. In fact, there are exams that include up to 1.9 images per clinical case associated with an image. Once again, like the issue with characters, including excessive information unrelated to the question increases difficulty, confuses candidates, and reduces discriminatory power. To improve this discriminatory power, it is probably necessary for image-associated questions to lower their difficulty level. This can be achieved by ensuring that the displayed image represents, without any doubt or interpretation, a useful and straightforward pathological finding for diagnosing the presented disease. It would be even better if the concept being asked directly relates to the semiotics of the image and avoids introducing complications associated with long and subjective answer choices.

Unfortunately, we did not collect data on the subjectively perceived difficulty of the questions as this aspect was not within the scope of our study. It could be very interesting to evaluate in future studies.

## Conclusions

In summary, for a test question within the context of the MIR exam and potentially applicable to the broader medical and healthcare field, high-quality questions would be those that: The best approach is to inquire using a clinical case format, followed by direct short-answer test questions. Have low difficulty to avoid concepts that are ambiguous or based on limited scientific evidence, which could lead to interpretation errors among the strong group of exam takers. Provide a clear and specific answer based on appropriate scientific evidence, avoiding ambiguous problem cases. Present typical clinical cases of the disease with its key characteristic features, avoiding the fuzzy boundaries of medical knowledge. Have a lengthy stem that includes all the necessary information for diagnosis without contradictions. Have very brief and specific answer choices, avoiding speculation that may lead knowledgeable students astray. If associated with images, the images should be typical and clear, consistent with the rest of the examination, and presented within a clinical case with clinical semiotics and propaedeutics. These criteria contribute to creating high-quality test questions that promote accurate assessment, minimize ambiguity, and maximize discriminatory power in medical and healthcare settings.

## Data Availability

The datasets used and/or analysed during the current study available from the corresponding author on reasonable request.
